# Supplementary material for: Influence of Metal Cations on the Viscoelastic Properties of Escherichia coli Biofilms
Source: ACS Omega. 2023 Jan 27;8(5):4667–76. doi: 10.1021/acsomega.2c06438 (PMC9910073; doi:10.1021/acsomega.2c06438)
Supplement: Supplementary file 1 — ao2c06438_si_001.pdf [file ao2c06438_si_001.pdf]

Supporting Information

Influence of metal cations on the viscoelastic properties of *Escherichia coli* biofilms

Adrien Sarlet[1], Valentin Ruffine[2], Kerstin G. Blank[2,3*], Cécile M. Bidan[1*]

[1]Max Planck Institute of Colloids and Interfaces, Department of Biomaterials, Am Mühlenberg 1, 14476 Potsdam, Germany

[2]Max Planck Institute of Colloids and Interfaces, Mechano(bio)chemistry, Am Mühlenberg 1, 14476 Potsdam, Germany

[3]Johannes Kepler University, Institute of Experimental Physics, Altenberger Str. 69, 4040 Linz, Austria

Corresponding authors:

Kerstin G. Blank: kerstin.blank@jku.at

Cécile M. Bidan: cecile.bidan@mpikg.mpg.de

Table of Contents

1. Osmolality measurement 3

2. Amplitude sweeps for AR3110 samples with ascending and descending strain amplitude 3

3. Frequency sweeps for AR3110 samples prepared under different conditions 4

4. Effect of diluting biofilm samples with ultrapure water 7

5. Dilution experiments with metal cation solutions...7

6. Counter ion condensation 14

7. References 16

1. **Osmolality measurement**

NaCl solutions, spanning the concentration range from 200-550 mM, were prepared as three independent replicates for each point. The osmolalities of these standard solutions were measured, a linear regression was fitted using the least squares method with the *lm* function in R (R Core Team; version 4.0.5) and the resulting calibration curve was plotted. Subsequently, the osmolality of all used salt solutions (220 mM) was determined from three aliquots of the same solution. For the osmolality control solution used for biofilm experiments, a NaCl solution was prepared with a concentration (409 mM) that matched the osmolality of the 220 mM $FeCl_3$ solution of 754 mOsm kg^{-1} (Figure S1).

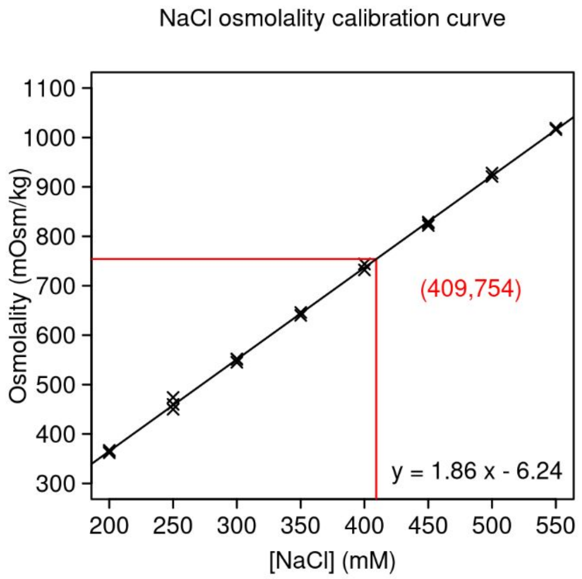

Figure S1. Osmolality calibration curve obtained with NaCl solutions of different concentrations. The 220 mM $FeCl_3$ solution has an osmolality of 754 mOsm kg^{-1}. This corresponds to a NaCl concentration of 409 mM.

2. **Amplitude sweeps for AR3110 samples with ascending and descending strain amplitude**

Amplitude sweeps were performed for AR3110 biofilms with and without the addition of 10 % (v/w) ultrapure water (Figure S2). The oscillation frequency was set to 10 rad s^{-1}. The strain amplitude was increased from 0.01 % to 100 % with 7 points per decade and then decreased again. These cycles of ascending and descending strain amplitude were repeated 3x. One experiment with 3 cycles lasted approximately 45 min.

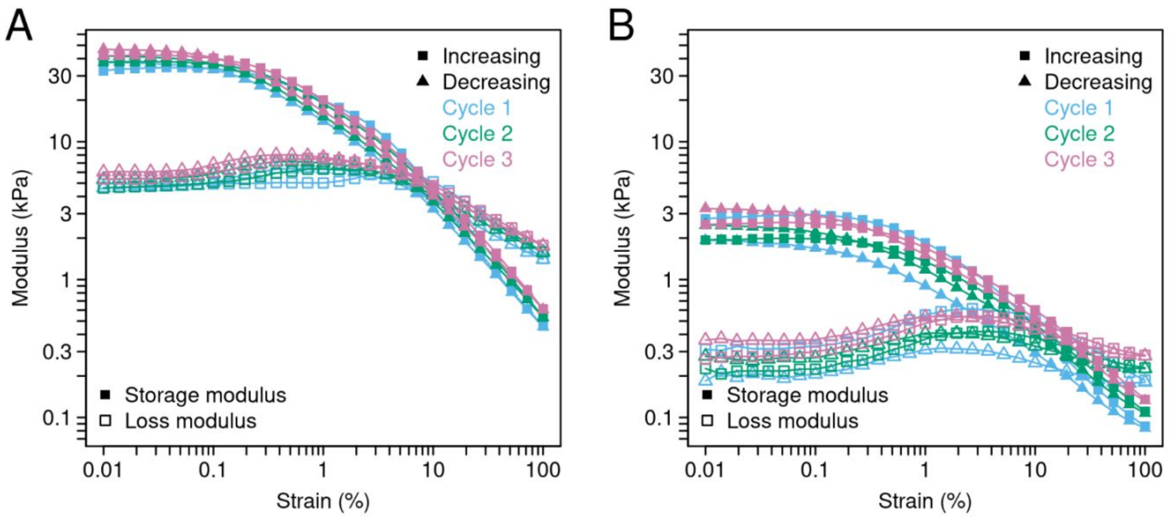

Figure S2. Amplitude sweeps of the strain AR3110. A) Amplitude sweep of a neat sample, consisting of three cycles of ascending and descending strain amplitudes. B) Amplitude sweep of a sample diluted with 10 % (v/w) ultrapure water, consisting of three cycles of ascending and descending strain amplitudes. The amplitude sweeps were performed at a frequency of 10 rad s^{-1}.

3. Frequency sweeps for AR3110 samples prepared under different conditions

Frequency sweeps were performed for AR3110 samples (1) with and without the addition of 10 % (v/w) ultrapure water, (2) preceded or not by an amplitude sweep or (3) starting from the highest (descending) or the lowest (ascending) frequency. The applied strain (γ = 0.02 %) was determined from the linear viscoelastic range of the amplitude sweeps (Figure S2). The frequency range of two decades was centred on the frequency used for the amplitude sweeps (ω = 10 rad s^{-1}) (Figure S3).

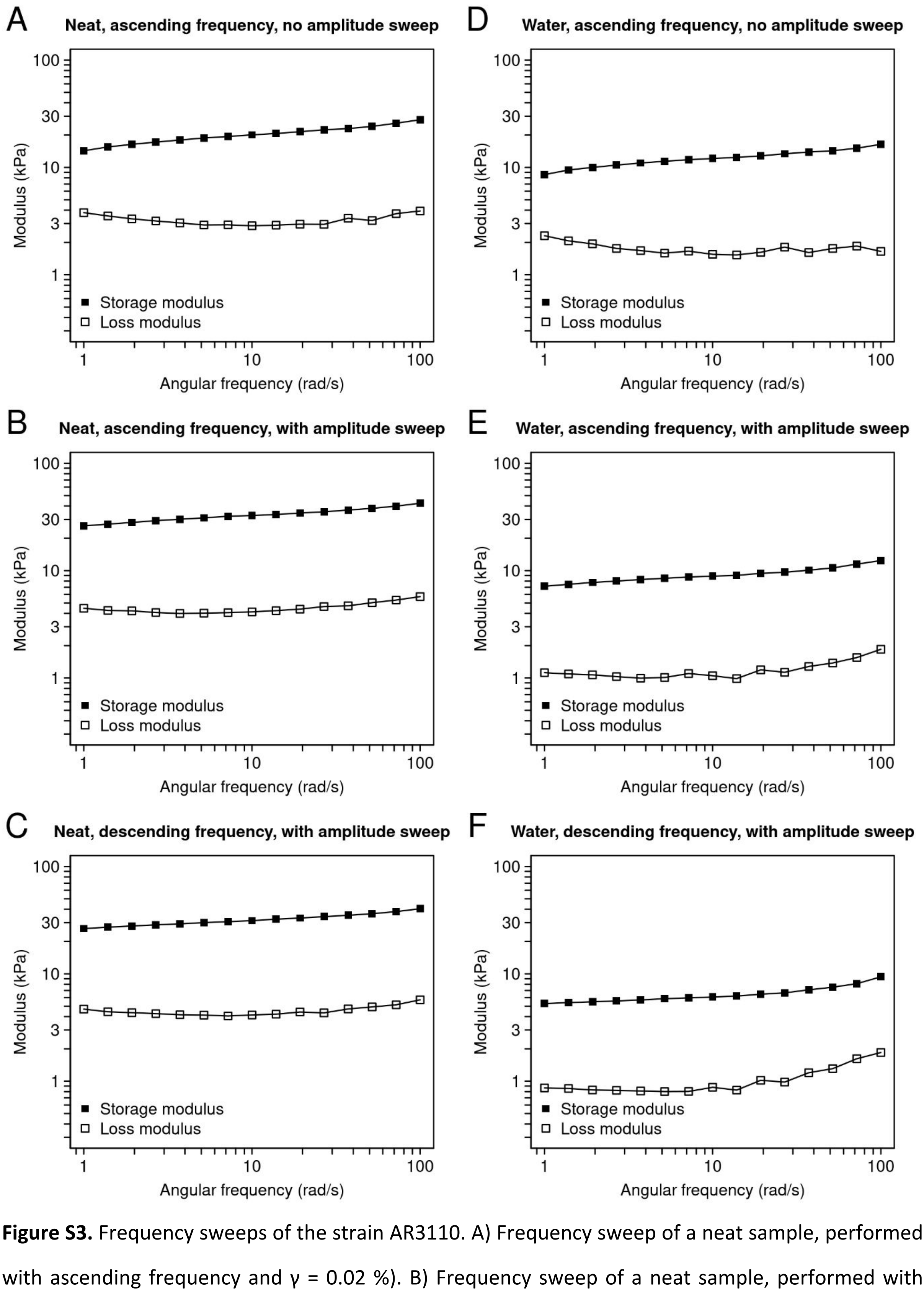

Figure S3. Frequency sweeps of the strain AR3110. A) Frequency sweep of a neat sample, performed with ascending frequency and $\gamma = 0.02$ %). B) Frequency sweep of a neat sample, performed with ascending frequency and $\gamma = 0.02$ %. Before the frequency sweep, the sample was subjected to one cycle of ascending and descending strain amplitudes. C) Frequency sweep of a neat sample, performed

with descending frequency and γ = 0.02 %. Before the frequency sweep, the sample was subjected to one cycle of ascending and descending strain amplitudes. D) Frequency sweep of a sample diluted with 10 % (v/w) ultrapure water. The frequency sweep was performed with ascending frequency and γ = 0.02 %. E) Frequency sweep of a sample diluted with 10 % (v/w) ultrapure water. The frequency sweep was performed with ascending frequency and γ = 0.02 %. Before the frequency sweep, the sample was subjected to one cycle of ascending and descending strain amplitudes. F) Frequency sweep of a sample diluted with 10 % (v/w) ultrapure water. The frequency sweep was performed with descending frequency and γ = 0.02 %. Before the frequency sweep, the sample was subjected to one cycle of ascending and descending strain amplitudes.

In addition, a frequency sweep was performed over five decades (ω = 0.001-100 rad s^{-1}), using a neat or diluted sample (with preceding amplitude sweep and descending strain amplitude). The measurement was performed to determine the relaxation time of the biofilm samples; however, drying of the samples significantly affected the data in the low frequency range (Figure S4).

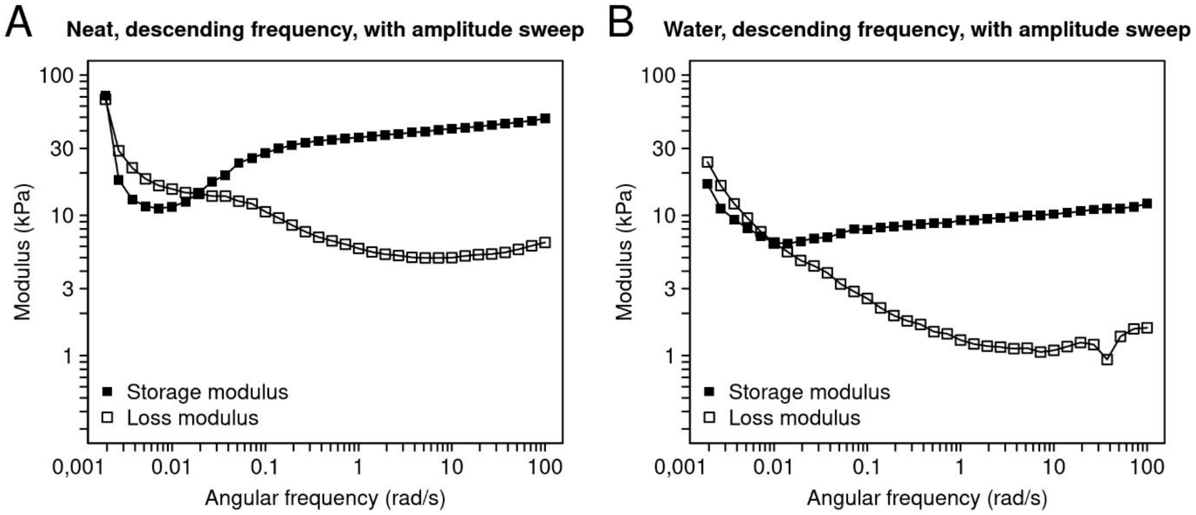

Figure S4. Extended frequency sweeps of the strain AR3110. A) Frequency sweep of a neat sample, performed with descending frequency and γ = 0.02 %. Before the frequency sweep, the sample was subjected to one cycle of ascending and descending strain amplitudes. B) Frequency sweep of a sample diluted with 10 % (v/w) ultrapure water. The frequency sweep was performed with descending frequency and γ = 0.02 %. Before the frequency sweep, the sample was subjected to one cycle of ascending and descending strain amplitudes.

4. **Effect of diluting biofilm samples with ultrapure water**

To determine the effect of adding 10 % (v/w) ultrapure water to the different biofilm samples, storage and loss moduli were extracted from the amplitude sweeps of ≥3 independent biofilm samples (see Methods). The extracted values, as well as their median values, are reported in Tables S1 and S2.

Table S1. Storage moduli (G'_0) of neat biofilms (-) and biofilms diluted (+) with 10 % (v/w) water.

**Matrix composition**	**Curli pEtN-cellulose**		**Curli**		**pEtN-cellulose**		**Curli pEtN-cellulose (mixed)**		**Curli pEtN-cellulose (co-seeded)**	
**Water**	**-**	**+**	**-**	**+**	**-**	**+**	**-**	**+**	**-**	**+**
	37933	1940	27500	2703	18200	576	27000	1423	54400	8333
	24400	2933	10017	1213	9157	190	31267	2673	24067	2857
G'_0 (Pa)	28267	5373	16533	1580	30567	1057	45200	6797	51167	5617
	32833	4510								
	20367	4613								
**Median**	**28267**	**4510**	**16533**	**1580**	**18200**	**576**	**31267**	**2673**	**51167**	**5617**

Table S2. Loss moduli (G''_0) of neat biofilms (-) and biofilms diluted (+) with 10 % (v/w) water.

**Matrix composition**	**Curli pEtN-cellulose**		**Curli**		**pEtN-cellulose**		**Curli pEtN-cellulose (mixed)**		**Curli pEtN-cellulose (co-seeded)**	
**Water**	**-**	**+**	**-**	**+**	**-**	**+**	**-**	**+**	**-**	**+**
	4663	217	4360	399	2187	61	3413	134	6390	843
	3197	359	1390	156	1180	11	3137	225	3430	367
G''_0 (Pa)	3297	539	2047	170	3367	107	4923	654	6550	695
	4330	479								
	1950	442								
**Median**	**3297**	**442**	**2047**	**170**	**2187**	**61**	**3413**	**225**	**6390**	**695**

5. **Dilution experiments with metal cation solutions**

To determine the effect of adding solutions of metal cations to the different biofilm samples, storage and loss moduli were extracted from the amplitude sweeps of ≥5 independent biofilm samples (see Methods). The extracted values, as well as their median values, are reported in Tables S3 to S12.

Table S3. Storage moduli (G'$_0$) of **AR3110** biofilms incubated with 10 % (v/w) water or a solution of interest. Pairs of values located in the same line correspond to biofilms grown in the same Petri dish. Samples incubated with NaCl and HCl were grown in the same Petri dish, which is why they share the same water control values.

**Matrix composition**	**Curli pEtN-cellulose**											
**Solution**	**$AlCl_3$**	**Water**	**$FeCl_3$**	**Water**	**$ZnCl_2$**	**Water**	**$CaCl_2$**	**Water**	**NaCl**	**Water**	**HCl**	**Water**
	7100	3913	40100	23200	7097	14733	12067	6750	3997	9623	10733	9623
	13333	4893	38400	34333	11367	13767	12700	19167	1803	11000	8163	11000
	7520	2270	33133	24633	14267	20500	8800	21767	2603	12633	4400	12633
	5543	3460	32700	21400	11000	14800	11367	18933	608	1042	1187	1042
	6797	4927	38500	25367	8553	16633	16033	23033	2023	9537	4600	9537
G'$_0$ (Pa)	3193	2780	171000	52767	17467	16033	23733	20567				
	117667	19133										
	14567	4713										
	20600	5830										
	8073	3973										
	11033	6050										
**Q1**	**6948**	**3687**	**34450**	**23558**	**9165**	**14750**	**11542**	**18992**	**1803**	**9537**	**4400**	**9537**
**Q2 (median)**	**8073**	**4713**	**38450**	**25000**	**11183**	**15417**	**12383**	**19867**	**2023**	**9623**	**4600**	**9623**
**Q3**	**13950**	**5378**	**39700**	**32092**	**13542**	**16483**	**15200**	**21467**	**2603**	**11000**	**8163**	**11000**

Table S4. Loss moduli (G''_0) for **AR3110** biofilm samples incubated with 10 % (v/w) water or a solution of interest. Pairs of values located in the same line correspond to biofilms grown in the same Petri dish. Samples incubated with NaCl and HCl were grown in the same Petri dish, which is why they share the same water control values.

**Matrix composition**						**Curli pEtN-cellulose**						
**Solution**	**$AlCl_3$**	**Water**	**$FeCl_3$**	**Water**	**$ZnCl_2$**	**Water**	**$CaCl_2$**	**Water**	**NaCl**	**Water**	**HCl**	**Water**
	1043	587	5690	2820	1457	2067	2520	968	447	878	1067	878
	2187	794	5613	4397	2253	1653	2250	2613	242	1373	1127	1373
	1270	333	4763	2883	2613	2483	1553	2847	380	1770	583	1770
	977	517	4650	2513	2017	1860	2053	2360	99	158	114	158
	1277	979	5383	2990	1630	2487	3093	2993	278	1240	542	1240
G''_0 (Pa)	572	329	22800	6117	2987	1830	4803	3517				
	14633	2060										
	2073	563										
	2850	676										
	1203	582										
	1760	1227										
**Q1**	**1123**	**540**	**4918**	**2836**	**1727**	**1838**	**2103**	**2423**	**242**	**878**	**542**	**878**
**Q2 (median)**	**1277**	**587**	**5498**	**2937**	**2135**	**1963**	**2385**	**2730**	**278**	**1240**	**583**	**1240**
**Q3**	**2130**	**886**	**5671**	**4045**	**2523**	**2379**	**2950**	**2957**	**380**	**1373**	**1067**	**1373**

Table S5. Storage moduli (G'$_0$) for **W3110** biofilms incubated with 10 % (v/w) water or a solution of interest. Pairs of values located in the same line correspond to biofilms grown in the same Petri dish.

**Matrix composition**	**Curli**							
**Solution**	**$AlCl_3$**	**Water**	**$FeCl_3$**	**Water**	**$ZnCl_2$**	**Water**	**$CaCl_2$**	**Water**
G'$_0$ (Pa)	3550	8737	3287	6630	6427	20967	3900	10133
	21400	9613	9393	11000	2413	4793	3737	8587
	11133	10833	2247	5153	2857	10133	3210	14667
	10500	4973	8290	23067	4717	7167	5777	15367
	9940	6103	3350	5763	1323	5283	4050	10667
	12267	6543	4947	6000	4990	8360	11333	11933
**Q1**	**10080**	**6213**	**3302**	**5823**	**2524**	**5754**	**3777**	**10267**
**Q2 (median)**	**10817**	**7640**	**4148**	**6315**	**3787**	**7763**	**3975**	**11300**
**Q3**	**11983**	**9394**	**7454**	**9908**	**4922**	**9690**	**5345**	**13983**

Table S6. Loss moduli (G"$_0$) for **W3110** biofilms incubated with 10 % (v/w) water or a solution of interest. Pairs of values located in the same line correspond to biofilms grown in the same Petri dish.

**Matrix composition**	**Curli**							
**Solution**	**$AlCl_3$**	**Water**	**$FeCl_3$**	**Water**	**$ZnCl_2$**	**Water**	**$CaCl_2$**	**Water**
G"$_0$ (Pa)	584	2187	479	1507	1353	3287	710	2093
	4393	2433	2030	2650	481	939	714	1780
	2353	2677	351	1042	516	1780	599	2637
	2063	1253	1630	3467	920	1317	1083	2477
	1833	1697	548	1197	236	861	763	1880
	2327	1477	888	1140	955	1510	2513	2097
**Q1**	**1891**	**1532**	**496**	**1154**	**490**	**1034**	**711**	**1933**
**Q2 (median)**	**2195**	**1942**	**718**	**1352**	**718**	**1413**	**739**	**2095**
**Q3**	**2347**	**2372**	**1445**	**2364**	**946**	**1713**	**1003**	**2382**

Table S7. Storage moduli (G'_0) for **AP329** biofilms incubated with 10 % (v/w) water or a solution of interest. Pairs of values located in the same line correspond to biofilms grown in the same Petri dish.

**Matrix composition**				**pEtN-cellulose**				
**Solution**	**$AlCl_3$**	**Water**	**$FeCl_3$**	**Water**	**$ZnCl_2$**	**Water**	**$CaCl_2$**	**Water**
G'_0 (Pa)	5337	3470	1353	1443	1977	3250	5137	11133
	2107	4933	1417	6847	1600	10367	4773	21267
	3530	9213	1623	8413	3153	16500	6960	18933
	9580	10800	6233	9103	2173	10600	4857	17233
	12533	17067	8857	16933	1203	8540	8577	14767
	7843	16567			1870	10233	8157	19800
	10100	4043						
	10767	7387						
**Q1**	**4885**	**4711**	**1417**	**6847**	**1668**	**8963**	**4927**	**15383**
**Q2 (median)**	**8712**	**8300**	**1623**	**8413**	**1923**	**10300**	**6048**	**18083**
**Q3**	**10267**	**12242**	**6233**	**9103**	**2124**	**10542**	**7857**	**19583**

Table S8. Loss moduli (G''_0) for **AP329** biofilms incubated with 10 % (v/w) water or a solution of interest. Pairs of values located in the same line correspond to biofilms grown in the same Petri dish.

**Matrix composition**				**pEtN-cellulose**				
**Solution**	**$AlCl_3$**	**Water**	**$FeCl_3$**	**Water**	**$ZnCl_2$**	**Water**	**$CaCl_2$**	**Water**
G''_0 (Pa)	707	345	179	152	375	370	1002	1397
	363	536	185	800	265	1220	933	2660
	571	1077	216	975	558	1970	1377	2450
	1913	1533	988	1190	380	1250	976	2297
	2450	2450	1377	2353	213	969	1800	2013
	1150	1980			334	1100	1680	2703
	1563	448						
	1680	862						
**Q1**	**673**	**514**	**185**	**800**	**282**	**1002**	**983**	**2084**
**Q2 (median)**	**1357**	**969**	**216**	**975**	**355**	**1160**	**1189**	**2373**
**Q3**	**1738**	**1645**	**988**	**1190**	**379**	**1243**	**1604**	**2608**

Table S9. Storage moduli (G'$_0$) for samples consisting of **mixed W3110 and AP329** biofilms incubated with 10 % (v/w) water or a solution of interest. Pairs of values located in the same line correspond to biofilms grown in the same Petri dish.

**Matrix composition**	**Curli pEtN-cellulose (mixed)**							
**Solution**	**$AlCl_3$**	**Water**	**$FeCl_3$**	**Water**	**$ZnCl_2$**	**Water**	**$CaCl_2$**	**Water**
G'$_0$ (Pa)	1950	15900	4107	20767	4153	9840	8053	19400
	20900	14833	17867	22167	4663	13000	7267	19733
	8627	6020	6640	10600	11467	29333	11800	47767
	6847	11400	15167	19833	8180	14933	6157	46500
	13167	28100	16933	21233	5023	36500	15033	57600
	11833	15700					11967	30400
**Q1**	**7292**	**12258**	**6640**	**19833**	**4663**	**13000**	**7463**	**22400**
**Q2 (median)**	**10230**	**15267**	**15167**	**20767**	**5023**	**14933**	**9927**	**38450**
**Q3**	**12833**	**15850**	**16933**	**21233**	**8180**	**29333**	**11925**	**47450**

Table S10. Loss moduli (G"$_0$) for samples consisting of **mixed W3110 and AP329** biofilms, incubated with 10 % (v/w) water or a solution of interest. Pairs of values located in the same line correspond to biofilms grown in the same Petri dish.

**Matrix composition**	**Curli pEtN-cellulose (mixed)**							
**Solution**	**$AlCl_3$**	**Water**	**$FeCl_3$**	**Water**	**$ZnCl_2$**	**Water**	**$CaCl_2$**	**Water**
G"$_0$ (Pa)	266	2863	582	3570	770	1757	1420	2983
	3170	2387	3110	4253	830	1850	1280	2690
	1250	818	912	1827	2130	5413	2493	7087
	969	1653	2360	3567	1603	2640	1060	6787
	2020	4257	2367	3840	870	6443	3230	8387
	1727	2567					2443	5707
**Q1**	**1039**	**1837**	**912**	**3567**	**830**	**1850**	**1315**	**3664**
**Q2 (median)**	**1488**	**2477**	**2360**	**3570**	**870**	**2640**	**1932**	**6247**
**Q3**	**1947**	**2789**	**2367**	**3840**	**1603**	**5413**	**2481**	**7012**

Table S11. Storage moduli (G'_0) for **co-seeded W3110 and AP329** biofilms incubated with 10 % (v/w) water or a solution of interest. Pairs of values located in the same line correspond to biofilms grown in the same Petri dish.

**Matrix composition**				**Curli pEtN-cellulose (co-seeded)**				
**Solution**	**$AlCl_3$**	**Water**	**$FeCl_3$**	**Water**	**$ZnCl_2$**	**Water**	**$CaCl_2$**	**Water**
G'_0 (Pa)	8263	7487	4540	9743	6423	15733	2890	19033
	16867	12100	2717	13833	8297	12500	8750	13233
	22300	20733	8023	20433	14500	17033	4700	16067
	24867	13667	25700	19400	7617	11367	5157	20500
	28167	27333	16200	18833	16933	21200	14867	13733
	19267	19500	21767	26167	5650	29200	3073	17900
**Q1**	**17467**	**12492**	**5411**	**15083**	**6722**	**13308**	**3480**	**14317**
**Q2 (median)**	**20783**	**16583**	**12112**	**19117**	**7957**	**16383**	**4928**	**16983**
**Q3**	**24225**	**20425**	**20375**	**20175**	**12949**	**20158**	**7852**	**18750**

Table S12. Loss moduli (G''_0) for **co-seeded W3110 and AP329** biofilms incubated with 10 % (v/w) water or a solution of interest. Pairs of values located in the same line correspond to biofilms grown in the same Petri dish.

**Matrix composition**				**Curli pEtN-cellulose (co-seeded)**				
**Solution**	**$AlCl_3$**	**Water**	**$FeCl_3$**	**Water**	**$ZnCl_2$**	**Water**	**$CaCl_2$**	**Water**
G''_0 (Pa)	1133	900	679	1277	1060	2183	456	2570
	2433	1583	327	1687	1597	1673	1697	1747
	3807	2557	1227	2817	2847	2357	789	1973
	3557	1767	3770	2833	1410	1457	826	2860
	4257	3537	2693	2520	3277	2663	2950	1667
	2877	2480	3163	3370	927	3600	472	2167
**Q1**	**2544**	**1629**	**816**	**1895**	**1148**	**1801**	**551**	**1803**
**Q2 (median)**	**3217**	**2123**	**1960**	**2668**	**1503**	**2270**	**807**	**2070**
**Q3**	**3744**	**2537**	**3046**	**2829**	**2534**	**2587**	**1479**	**2469**

6. Counter ion condensation in pEtN-cellulose

To assess whether counter ion condensation could occur along the pEtN-cellulose fibres, we calculated the following Manning's criterion, taking into account the valence of the cation possibly involved. Counter ion condensation occurs when:

$$\xi = \frac{l_B}{b} > \xi_{crit} = 1/z \qquad ,^{1,2}$$

where *z* is the valence of the counter ion, *b* the length per unit of charge (i.e. the distance between two neighbouring charges along the pEtN-cellulose chain) and l_B the Bjerrum length with

$$l_B = \frac{e^2}{4\pi\varepsilon_0\varepsilon k_B T} \cong 0.712\ nm \text{ in water at 20°C,}$$

and *e* the elementary charge, *ε* the relative permittivity, ε_0 the electric constant, k_B the Boltzmann constant and *T* the temperature.

The distance between two glucose units is 0.515 nm (data for carboxymethycellulose),[3] and half of the glucoses are substituted on their carbon 6 with phosphoethanolamine.[4] Considering one negative charge per phosphate, we obtain

$$b = 2 * 0.515 = 1.030\ nm \text{ and } \xi = \frac{0.712}{1.030} = 0.691 > \frac{1}{z}$$

for bivalent and trivalent ions. This means that counter ion condensation along the pEtN-cellulose chain can take place for both bivalent and trivalent ions.

Table S13. Quantities required for computing Manning's criterion in the case of pEtN-cellulose.

Symbol	Quantity	Value
e	Elementary charge	$1.602\ 10^{-19}$ C
ε_0	Vacuum permittivity	$8.854\ 10^{-12}$ $F.m^{-1}$
ε	Relative permittivity (water, 20°C)	80.08
k_B	Boltzmann constant	1.381 $J.K^{-1}$
T	Absolute temperature	293.15 K
b	Axial distance per elementary charge	1.030 nm

7. References

(1) Manning, G. S. Limiting Laws and Counterion Condensation in Polyelectrolyte Solutions I. Colligative Properties. *J. Chem. Phys.* **1969**, *51* (3), 924–933. https://doi.org/10.1063/1.1672157.

(2) Manning, G. S. Counterion Condensation on Charged Spheres, Cylinders, and Planes. *J. Phys. Chem. B* **2007**, *111* (29), 8554–8559. https://doi.org/10.1021/jp0670844.

(3) Zhivkov, A. M. Electric Properties of Carboxymethyl Cellulose. In *Cellulose - Fundamental Aspects*; InTech, 2013. https://doi.org/10.5772/56935.

(4) Thongsomboon, W.; Serra, D. O.; Possling, A.; Hadjineophytou, C.; Hengge, R.; Cegelski, L. Phosphoethanolamine Cellulose: A Naturally Produced Chemically Modified Cellulose. *Science (80-.).* **2018**, *359* (6373), 334–338. https://doi.org/10.1126/science.aao4096.

(5) Dunbar, M.; DeBenedictis, E.; Keten, S. Dimerization Energetics of Curli Fiber Subunits CsgA and CsgB. *npj Comput. Mater.* **2019**, *5* (1), 27. https://doi.org/10.1038/s41524-019-0164-5.

(6) DeBenedictis, E. P.; Ma, D.; Keten, S. Structural Predictions for Curli Amyloid Fibril Subunits CsgA and CsgB. *RSC Adv.* **2017**, *7* (76), 48102–48112. https://doi.org/10.1039/C7RA08030A.

(7) Kohonen, M. M.; Karaman, M. E.; Pashley, R. M. Debye Length in Multivalent Electrolyte Solutions. *Langmuir* **2000**, *16* (13), 5749–5753. https://doi.org/10.1021/la991621c.
